# Identification of heat shock protein 32 (Hsp32) as a novel target in acute lymphoblastic leukemia

**DOI:** 10.18632/oncotarget.1805

**Published:** 2014-03-04

**Authors:** Sabine Cerny-Reiterer, Renata A. Meyer, Harald Herrmann, Barbara Peter, Karoline V. Gleixner, Gabriele Stefanzl, Emir Hadzijusufovic, Winfried F. Pickl, Wolfgang R. Sperr, Junia V. Melo, Hiroshi Maeda, Ulrich Jäger, Peter Valent

**Affiliations:** ^1^ Ludwig Boltzmann Cluster Oncology, Medical University of Vienna, Austria; ^2^ Department of Internal Medicine I, Division of Hematology & Hemostaseology, Medical University of Vienna, Austria; ^3^ Department of Companion Animals and Horses, Clinic for Internal Medicine and Infectious Diseases, University of Veterinary Medicine Vienna, Vienna, Austria; ^4^ Institute of Immunology, Medical University of Vienna, Austria; ^5^ Department of Haematology Centre for Cancer Biology, Adelaide, Australia; ^6^ Laboratory of Microbiology and Oncology, Faculty of Pharmaceutical Sciences, Sojo University, Kumamoto and BioDynamics, Research Laboratory, Kumamoto, Japan

**Keywords:** ALL, imatinib-resistance, Hsp32, HO-1, targeting

## Abstract

Heat shock proteins (Hsp) are increasingly employed as therapeutic targets in oncology. We have shown that Hsp32, also known as heme oxygenase-1 (HO-1), serves as survival factor and potential target in Ph^+^ chronic myeloid leukemia. We here report that primary cells and cell lines derived from patients with acute lymphoblastic leukemia (ALL) express Hsp32 mRNA and the Hsp32 protein in a constitutive manner. Highly enriched CD34^+^/CD38^−^ ALL stem cells also expressed Hsp32. Two Hsp32-targeting drugs, pegylated zinc protoporphyrine (PEG-ZnPP) and styrene maleic acid-micelle-encapsulated ZnPP (SMA-ZnPP), induced apoptosis and growth arrest in the BCR/ABL1^+^ cell lines, in Ph^−^ lymphoblastic cell lines and in primary Ph^+^ and Ph^−^ ALL cells. The effects of PEG-ZnPP and SMA-ZnPP on growth of leukemic cells were dose-dependent. In Ph^+^ ALL, major growth-inhibitory effects of the Hsp32-targeting drugs were observed in imatinib-sensitive and imatinib-resistant cells. Hsp32-targeting drugs were found to synergize with imatinib, nilotinib, and bendamustine in producing growth inhibition and apoptosis in Ph^+^ ALL cells. A siRNA against Hsp32 was found to inhibit growth and survival of ALL cells and to synergize with imatinib in suppressing the growth of ALL cells. In conclusion, Hsp32 is an essential survival factor and potential new target in ALL.

## INTRODUCTION

Acute lymphoblastic leukemia (ALL) is a life-threatening neoplasm characterized by uncontrolled growth and leukemic expansion of immature lymphoblastic progenitor cells [1-4]. The prognosis and outcome in ALL depend on age, molecular defects and response to therapy [1-6]. In about 30% of all adult patients, leukemic cells display the Philadelphia chromosome (Ph) and the related fusion gene, *BCR/ABL1* [1-6]. In the ´pre-imatinib-era´, these patients had an extremely poor prognosis compared to patients with Ph^−^ ALL [5,6]. Since then the prognosis of patients with BCR/ABL1^+^ ALL has improved, which is largely attributable to the effects of novel BCR/ABL1-targeting drugs [7-12]. In fact, the BCR/ABL1 tyrosine kinase inhibitor (TKI) imatinib is effective in most patients with newly diagnosed Ph^+^ ALL, and sometimes even in patients with chemotherapy-resistant or relapsed Ph^+^ ALL, especially when applied in combination with conventional chemotherapy [7-13]. Second- and third generation BCR/ABL1 blockers are also effective in patients with Ph^+^ ALL [14].

However, not all patients with Ph^+^ ALL respond to standard treatment and TKI. Therefore, depending on age, risk factors, and availability of a donor, stem cell transplantation (SCT) is recommended for patients with drug-resistant and high risk ALL [15-18]. In these patients, the overall treatment plan often combines chemotherapy, SCT and BCR/ABL1-targeting drugs [17]. However, despite SCT and the availability of novel targeted drugs, not all patients with Ph^+^ ALL can be cured. Therefore, current research focuses on identifying new targets and drugs that can be employed in these patients and may improve outcome and survival in ALL the future.

One class of interesting new targets in oncology are heat shock proteins (Hsp). These proteins often act as survival factors and are expressed in neoplastic cells in a constitutive manner [19]. Heat shock protein 32 (Hsp32), also known as heme oxygenase-1 (HO-1), is a stress-related cytoprotective molecule that is expressed in normal and neoplastic cells, including myeloid leukemias [20-28]. In neoplastic cells, Hsp32 is considered to play a major role as an essential survival factor [22-29]. We have recently shown that Hsp32 (HO-1) is expressed in leukemic cells in Ph^+^ chronic myeloid leukemia (CML) and that Hsp32-targeting drugs produce growth arrest and apoptosis in leukemic cells [28,29].

In the present study, we show that Hsp32 is expressed in leukemic cells in Ph^+^ and Ph^−^ ALL, and that pharmacologic inhibitors of Hsp32 suppress the growth of imatinib-sensitive as well as imatinib-resistant ALL cells. Moreover, we show that drug combinations consisting of Hsp32 inhibitors and either BCR/ABL1 TKI or bendamustin, can produce synergistic growth-inhibitory effects in imatinib-resistant ALL cells.

## RESULTS

### ALL cells express Hsp32 mRNA and the Hsp32 protein

**Figure 1:**
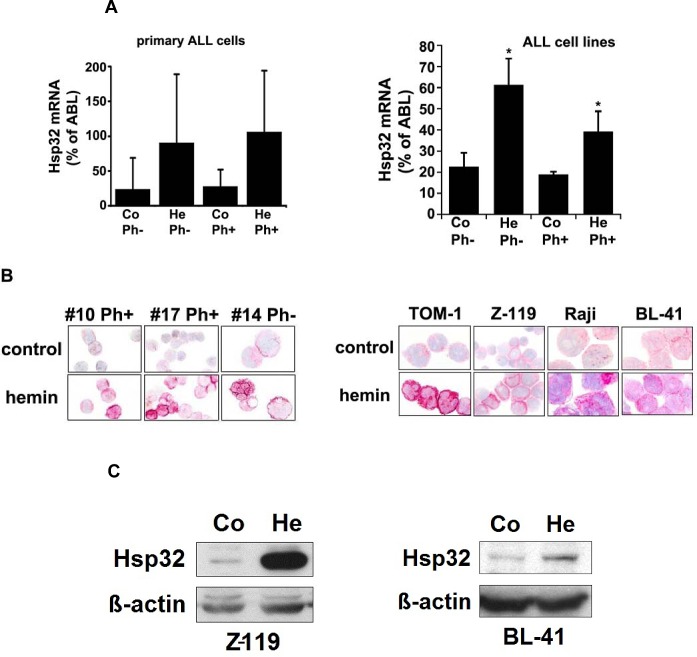

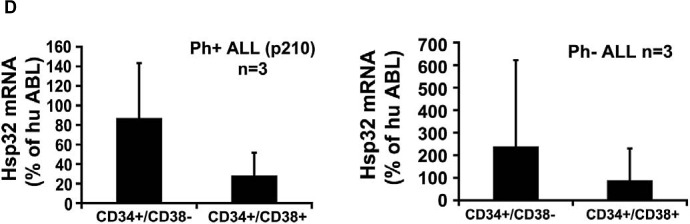
Expression of Hsp32 in ALL cells A: Primary ALL cells (left panel) and cell lines (right panel) were subjected to RNA isolation and RT-PCR using primers specific for Hsp32 and human ABL (control) as described in the text. Before RNA was isolated, cells were cultured in control medium (Co) or in medium containing 10 μM hemin (He) at 37°C for 8 hours. Expression of Hsp32 mRNA and ABL mRNA was determined by qPCR. The left panel shows data obtained with primary ALL cells (7 Ph^+^ donors and 10 Ph^─^ donors) and the right panel shows data obtained with Ph^+^ and Ph^─^ cell lines (Ph^+^: BV-173, NALM-1, TOM-1, Z-119, Ph^−^: Raji, Ramos, REH, BL-41). Hsp32 mRNA levels are expressed as percentage of ABL mRNA levels and represent the mean±S.D. from all donors or cell lines. Asterisk: p<0.05. B: Immunocytochemical detection of the Hsp32 protein in primary ALL cells (left panel, Ph^+^ patients #10 and #17; and Ph^−^ patient #14 from Table 1) and cell lines (right panel) after incubation in control medium or hemin (10 μM) at 37°C for 8 hours. After incubation, cells were spun on cytospin slides and stained with an antibody against Hsp32 as described in the text. Images were taken using an Olympus DP21 camera connected to an Olympus BX50F4 microscope equipped with 100x/1.35 UPlan-Apo objective lense (Olympus, Hamburg, Germany). Figures were prepared using Adobe Photoshop CS2 software version 9.0 (Adobe Systems, San Jose, CA) and processed with PowerPoint software (Microsoft, Redmond, WA). C: Western blot analysis of expression of Hsp32 in Ph^+^ cell line Z-119 (left) and Ph^─^ cell line BL-41 (right). Cells were incubated with control medium (Co) or hemin (10 μM) (He) at 37°C for 8 hours. Then, cells were lysed and lysates subjected to Western blot analysis using an antibody against Hsp32 and an antibody against ß-actin as described in the text. D: Expression of HO-1 mRNA in highly enriched (sorted) CD34^+^/CD38^−^ stem cells and CD34^+^/CD38^+^ progenitor cells obtained from 3 patients with Ph^−^ ALL (left side) and 3 patients with Ph^+^ ALL (p210 right side) as determined by qPCR. Cells were subjected to RNA isolation, cDNA synthesis and qPCR using primers specific for *HO-1* and *ABL*. Results show HO-1 mRNA levels as percent of ABL mRNA levels, and represent the mean±S.D. of 3 independent experiments (3 patients).

As assessed by qPCR, primary ALL cells as well as the ALL cell lines tested were found to express Hsp32 mRNA (Figure 1A, Tables 1 and 2). Hsp32 transcripts were present in Ph^+^ ALL cells as well as in Ph^−^ ALL cells (Figure 1A). Hemin was found to promote expression of Hsp23 mRNA in all ALL samples tested (Figure 1A). We were also able to show that ALL cells display the Hsp32 protein. Expression of the Hsp32 protein was demonstrable by immunocytochemistry (Figure 1B) as well as by Western blotting (Figure 1C), and hemin was found to upregulate expression of the Hsp32 protein in ALL cells (Figure 1B and 1C). Since leukemic stem cells are considered a major target of therapy, we were also interested to know whether CD34+/CD38− stem cells in ALL express Hsp32. In these experiments, we were able to show that highly enriched (sorted) CD34^+^/CD38^−^ ALL stem cells as well as CD34^+^/CD38^+^ progenitor cells express Hsp32 mRNA in patients with Ph^+^ ALL and patients with Ph^−^ ALL (Figure 1D).

**Table 1:** Patients´ characteristics, detection of Hsp32 in leukemic cells, and response to SMA-ZnPP and PEG-ZnPP

Patient No.(#)	Gender	Age (year)	Diagnosis	BCR/ABL1	WBC (G/L)	Hb (g/dL)	Plt (G/L)	Cytogenetics	ICC Hsp32	PCR Hsp32	SMA ZnPP IC50	PEG ZnPP IC50
#1	f	33	c-ALL	-	52.1	12.6	139	46,XX	+	+	20 μM	20 μM
#2	m	73	pre-B-ALL	p210	140	10	19	47,XY,t(9;22) (q34;q11)+20	+	+	5 μM	20 μM
#3	m	39	c-ALL	p190	61.6	9.4	56	46,XY, t(9;22) (q34;q11)	+	+	1 μM	20 μM
#4	f	20	pre-B-ALL	-	14.6	8.7	29	46,XX, complex	+	+	5 μM	n.t.
#5	f	35	c-ALL	p190	24.72	13.1	177	53,XX,t(9;22) (q34;q11, complex	+	+	1 μM	5 μM
#6	f	65	pre-B-ALL	-	170	9.1	111	46,XX,t(2;5), t(4;11)	+	+	n.t.	n.t.
#7	f	21	c-ALL	-	568	7.1	75	46,XX	+	+	1 μM	5 μM
#8	m	17	pre-T-ALL	-	4.2	8.1	16	47,XY, del(13), +19	+	+	n.t.	n.t.
#9	f	56	pre-B-ALL	p190	2.3	7.4	86	46,XX, t(9;22) (q34;q11)	+	+	n.t.	n.t.
#10	f	64	biphenotypic AL	p210	53.2	10.5	91	46,XX, t(9;22) (q34;q11)	+	+	n.t	n.t.
#11	f	71	T-ALL	-	9.3	5.5	30	46,XX, complex	+	+	10 μM	0.5 μM
#12	m	60	pre-B-ALL	-	37.6	18.5	77	46,XY, t(1;19),del(13)	+	+	5 μM	10 μM
#13	f	55	c-ALL	p190	71.29	13.7	73	46, XX, t(9;22) (q34;q11)	+	+	n.t.	n.t.
#14	m	17	pre-B-ALL	-	19.74	3.9	114	46,XY,del(9) (q21), idem+8	+	+	n.t.	n.t.
#15	m	60	c-ALL	p210	2.31	9	209	46,XY, t(9;22) (q34;q11), complex	+	+	0.5 μM	0.5 μM
#16	f	45	T-ALL	-	92.02	9	15	46,XX	+	+	n.t	n.t.
#17	f	35	c-ALL*	p210*	32.97	9.7	172	46,XX, t(9;22) (q34;q11)	+	+	5 μM	5 μM
#18	f	35	pre-B-ALL	-	155.26	8.6	52	46,XX,t(19;11), complex	+	+	n.t.	n.t.
#19	f	37	pre-B-ALL	p190	4.4	7.6	3	46,XX, t(9;22) (q34;q11)	+	+	n.t.	n.t.
#20	m	38	T-ALL	-	26.8	8.9	52	46,XY[13]/47, XY, del(13)(q14), der(14)x2, add(16)(q24)[2]	+	+	n.t.	n.t.
#21	f	57	pre-B-ALL	-	10.31	9.7	141	46,XX	n.t	n.t.	1 μM	5 μM
#22	m	21	biphenotypic AL	-	31.73	11.3	75	46,XY, t(2;14)	n.t.	n.t.	1 μM	20 μM
#23	m	63	CML, BP-Ly*	p210*	69.99	12.1	121	46,XY, t(9;22) (q34;q11), complex	n.t	n.t.	10 μM	1 μM
#24	f	22	relapsed pre-B-ALL	-	17.65	9.6	24	47,XX, t(14;14), complex	+	+	1 μM	n.t.
#25	m	72	c-ALL	p210	106.01	10.1	73	46, XY, t(9;22) (q34;q11)	n.t.	+	n.t.	n.t.
#26	f	32	pre-B-ALL	-	117.88	8.9	69	46,XX,del(9) (p13)	n.t.	+	n.t.	n.t.

* In these patients, imatinib resistance developed during the course of disease; in patient #23, a BCR/ABL1 T315I mutation was detected. For evaluation of proliferation, ALL cells were cultured in control medium or in various concentrations of Hsp32-inhibitors for 48 hours. Thereafter, 3H-thymidine-uptake was measured and IC50 values (μM) determined. Abbreviations: WBC, white blood cell count; Hb, hemoglobin; Plt, platelet count; ICC, immunocytochemistry; n.t., not tested; p210, BCR/ABL1 major breakpoint; p190, BCR/ABL1 minor breakpoint.

**Table 2:** Characterization of cell lines and response to SMA-ZnPP, PEG-ZnPP, and other drugs

Cell Line	Diagnosis	BCR- ABL1	Proliferation Imatinib IC50	ICC Hsp32	PCR Hsp32	Proliferation IC50		Apoptosis EC50	
						SMA-ZnPP IC50	PEG-ZnPP IC50	SMA-ZnPP EC50	PEG-ZnPP EC50
BV-173	B-ALL	p210	0.1 μM	+	+	1-5 μM	1-5 μM	5-10 μM	5 μM
NALM-1	CML, lymphoid BP	p210	0.4μM	+	+	1-5 μM	1-5μM	5 μM	n.t.
TOM-1	B-ALL	p190	0.3 μM	+	+	1-5 μM	1-5 μM	10 μM	15-20 μM
Z-119	B-ALL	p190	0.2 μM	+	+	1 μM	1-5 μM	5 μM	10 μM
RAJI	Burkitt lymphoma	-	>1 μM	+	+	5 μM	5 μM	5-10 μM	20 μM
RAMOS	Burkitt lymphoma	-	>1 μM	+	+	5 μM	5 μM	10 μM	20 μM
REH	B-ALL	-	>1 μM	+	+	5 μM	10 μM	5-10 μM	20 μM
BL-41	Burkitt lymphoma	-	>1 μM	+	+	1-5 μM	1-5 μM	1-5 μM	15 μM

CML, chronic myeloid leukemia; BP, blast phase; ALL, acute lymphoblastic leukemia; p210, BCR/ABL1 major breakpoint; p190, BCR/ABL1 minor breakpoint. Immunocytochemical (ICC) analysis of Hsp32 (HO-1) and qPCR analysis of Hsp32 (HO-1) mRNA expression were performed as described in the text. Cell lines were cultured in control medium or in various concentrations of the Hsp32 (HO-1) inhibitors SMA-ZnPP or PEG-ZnPP, or in the presence of various concentrations of the BCR/ABL1 kinase blockers imatinib or nilotinib for 48 hours. Thereafter, proliferation was measured by 3H-thymidine incorporation assay. IC50 values (μM) represent the mean from at least 3 independent experiments. Apoptosis was determined by light microscopy; EC50 values (μM) represent the mean from at least 3 independent experiments. n.t., not tested.

### BCR/ABL1-targeting drugs down regulate expression of Hsp32 in ALL cells

**Figure2:**
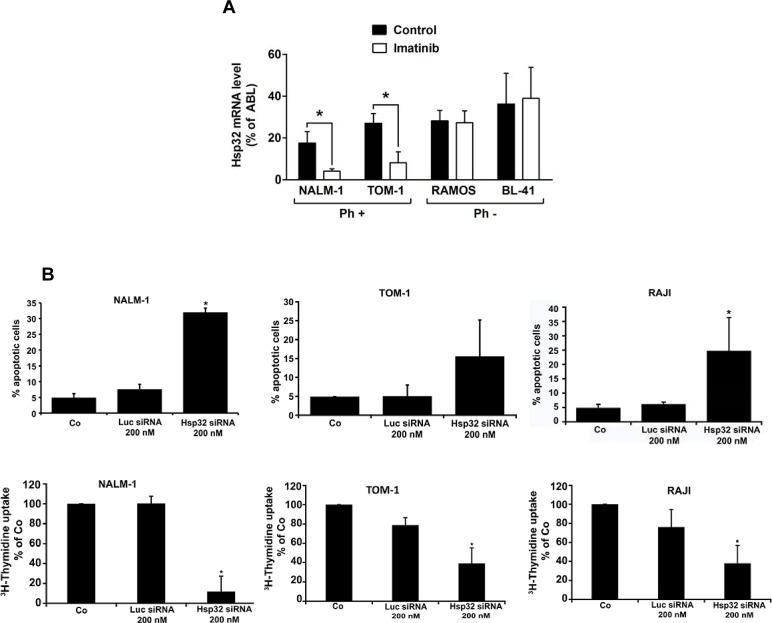

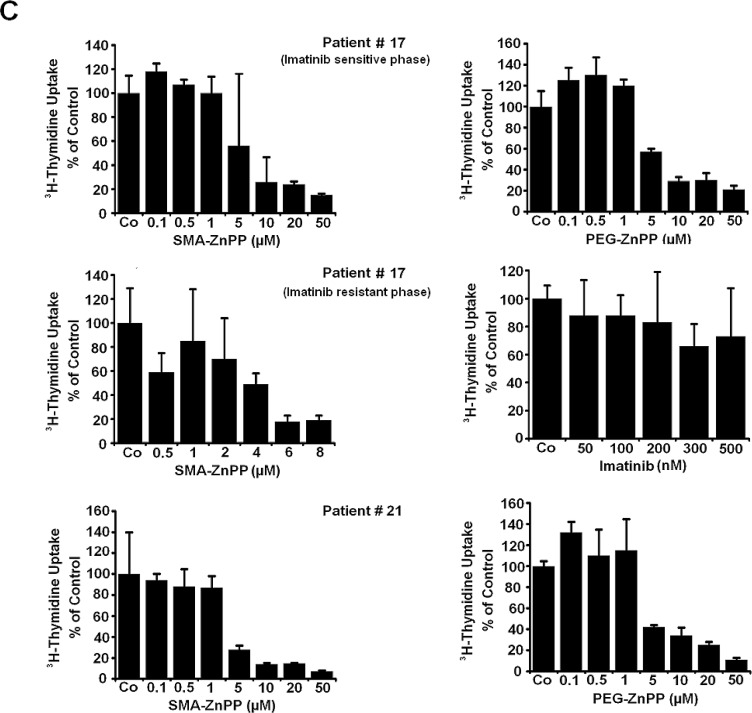

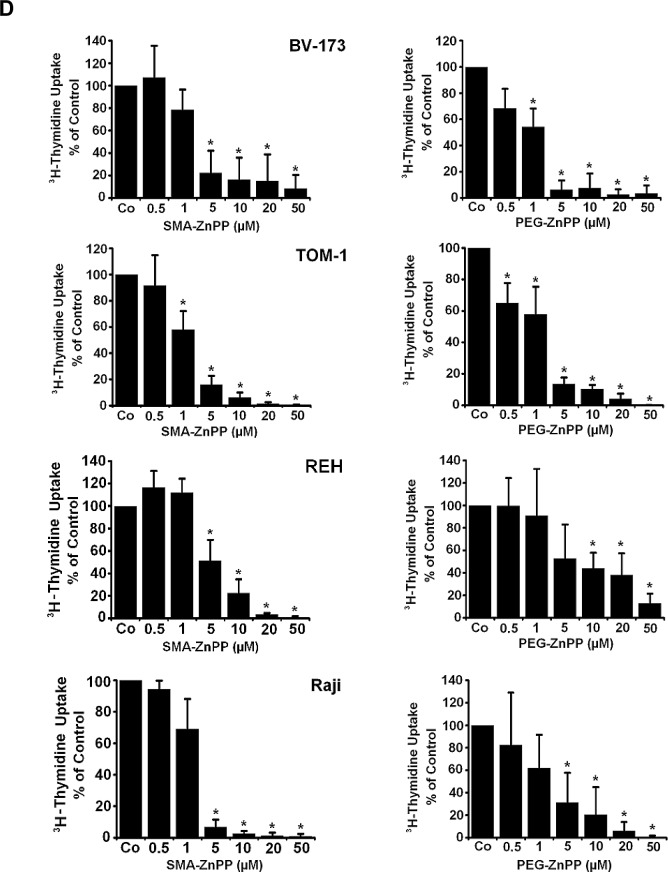
Targeting of Hsp32 in ALL cells and functional consequences A: Effects of BCR/ABL1-targeting drugs on expression of Hsp32 mRNA in leukemic cells. The Ph^+^ ALL cell lines NALM-1 and TOM-1 and the Ph^─^ cell line Ramos and BL-41 were incubated in control medium or imatinib (1 μM) at 37°C for 24 hours. Then, cells were recovered and subjected to RNA isolation and real time PCR using primers specific for Hsp32 and ABL. Results show Hsp32 mRNA expression levels relative to ABL mRNA levels. B: siRNA-induced Hsp32 knockdown in ALL cells is followed by apoptosis. The ALL cell lines NALM-1, TOM-1 and Raji were kept in control medium (Co) or were transfected with a control siRNA against luciferase (Luc siRNA) or siRNA against Hsp32 (200 nM each) as described in the text. After 48 hours, the percentage of apoptotic cells was determined by light microscopy (upper panel). Results represent the mean±S.D. from three independent experiments. Lower panel: ^3^H-thymidine uptake was measured after transfection with control siRNA or siRNA against Hsp32. Results show the percent of ^3^H-thymidine uptake compared to control and are expressed as mean±S.D. of triplicates. C,D: PEG-ZnPP and SMA-ZnPP inhibit growth of ALL cells. Primary leukemic cells (C) were obtained from patient #17 at two different time points, i.e. when cells were imatinib-sensitive and later when the patient developed imatinib-resistance, and from patient #21 with Ph^−^ ALL. Cells were incubated in control medium (Co), various concentrations of PEG-ZnPP or various concentrations of SMA-ZnPP (as indicated) at 37°C for 48 hours. In patient #17, cells were also incubated with imatinib (0.05-0.5 μM) for 48 hours. After incubation, ^3^H-thymidine uptake was measured. Results show the percentage of ^3^H-thymidine uptake compared to control and are expressed as mean±S.D. of triplicates. Figure 2D shows result obtained with the Ph^+^ ALL cell lines BV-173 and TOM-1 and the Ph^─^ cell lines REH and Raji. Results show the percentage of ^3^H-thymidine uptake compared to control and are expressed as mean±S.D. of three independent experiments. Asterisk indicates p<0.05.

We have recently shown that expression of Hsp32 in CML cells is triggered by BCR/ABL1 [28,29]. Therefore, we were interested to learn whether BCR/ABL1-targeting drugs would alter expression of Hsp32 in Ph^+^ ALL cells. As assessed by qPCR, imatinib was found to down regulate expression of Hsp32 mRNA in the Ph^+^ ALL cell lines TOM-1 and NALM-1 (Figure 2A). In contrast, imatinib did not suppress expression of Hsp32 mRNA in the Ph^−^ ALL cell lines tested (Figure 2A). These data suggest that BCR/ABL1 is involved in the expression of Hsp32 in Ph^+^ ALL, whereas in Ph^−^ ALL, other mechanisms contribute to Hsp32 expression.

### Depletion of Hsp32 leads to apoptosis and growth arrest in ALL cells

Hsp32 is a well-known survival factor that counteracts apoptosis in various cell types. To investigate the functional role of Hsp32 in ALL cells, expression of Hsp32 was specifically silenced by siRNA in the Ph^+^ ALL cell lines TOM-1 and NALM-1 and in the Ph^−^ cell line Raji. The siRNA-induced knockdown of Hsp32 was found to be associated with a significant decrease in cell viability due to an increase of apoptotic cells (Figure 2B). A control siRNA (against Luc) showed no substantial effect on expression of Hsp32 and no effect on survival (apoptosis) of ALL cells (Figure 2B). As expected, the siRNA-induced knock-down of Hsp32 also induced growth arrest in the ALL cell lines tested (Figure 2B).

### Effects of pharmacologic inhibitors of Hsp32 on growth of ALL cell lines

To evaluate the role of Hsp32 as a potential therapeutic target in ALL cells, two water-soluble pharmacologic inhibitors were applied, SMA-ZnPP and PEG-ZnPP. As assessed by ^3^H-thymidine uptake, both Hsp32-targeting drugs were found to inhibit the proliferation of Ph^+^ and Ph^−^ ALL cells (primary cells and cell lines) after 48 hours of incubation (Figure 2C and Figure 2D). The effects of both drugs on growth of ALL cells were dose-dependent, with comparable IC50 values (Tables 1 and 2).

### Hsp32-targeting drugs suppress the growth of leukemic cells from patients with imatinib-resistant ALL

In a substantial number of patients with ALL, leukemic cells develop resistance to imatinib. We were therefore interested to know whether Hsp32-targeting drugs can suppress the growth of leukemic cells from patients with imatinib-resistant Ph^+^ ALL. In these experiments, SMA-ZnPP and PEG-ZnPP were found to inhibit growth of primary, imatinib-resistant leukemic cells in a dose-dependent manner in all patients examined (Figure 2C, Table 1), including one patient with lymphoid blast phase exhibiting BCR/ABL T315I (Table 1).

### The growth-inhibitory effects of PEG-ZnPP and SMA-ZnPP on ALL cells are associated with induction of apoptosis

**Figure3:**
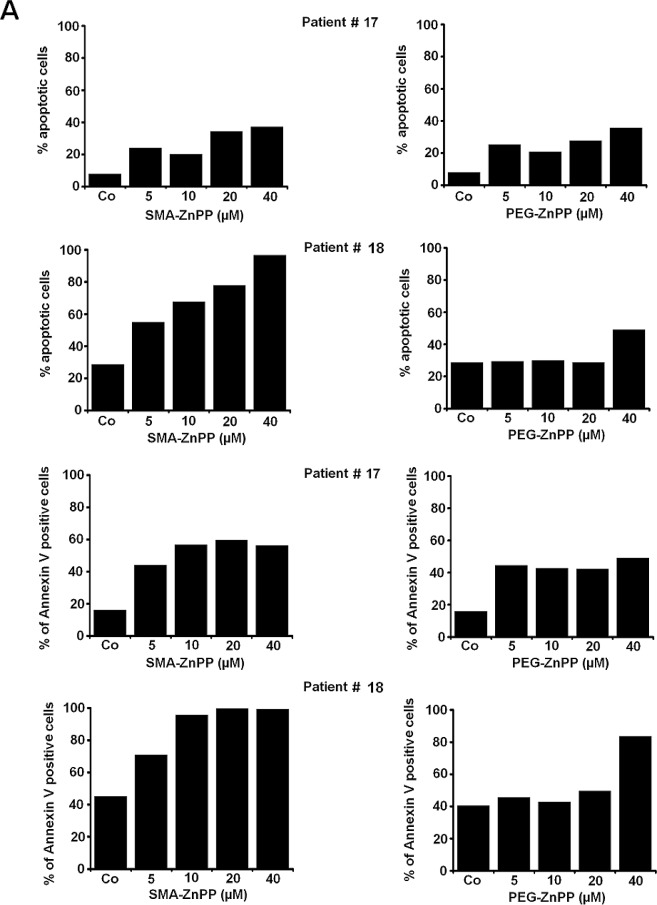

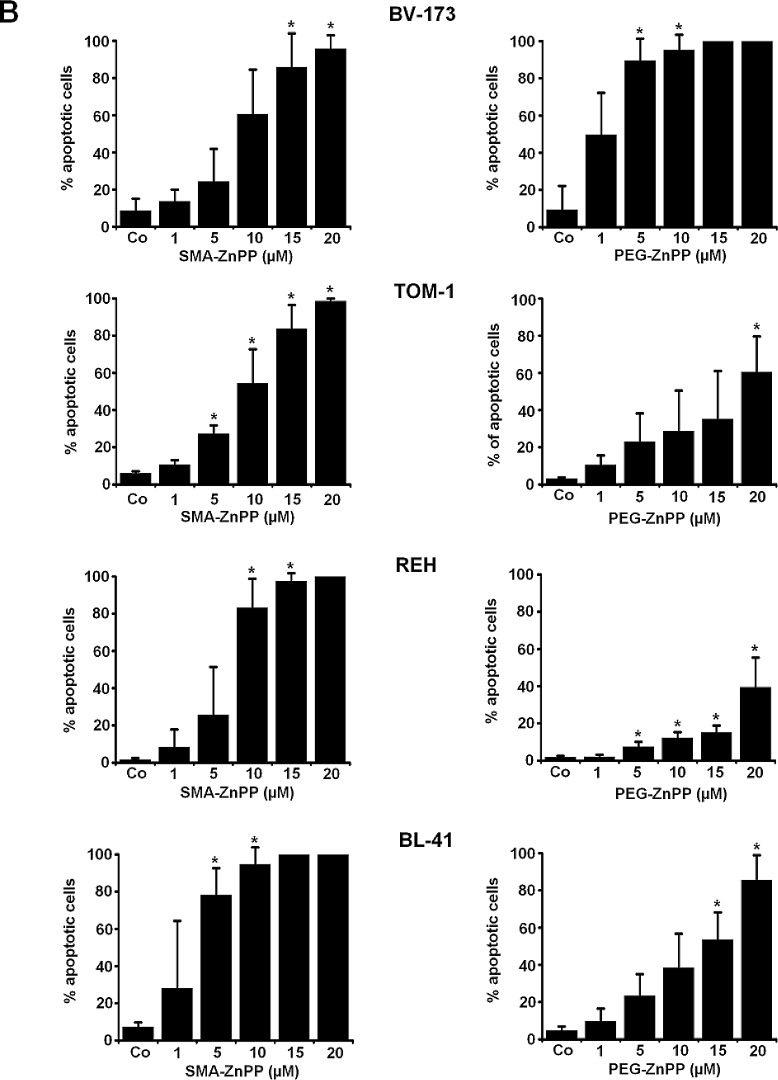

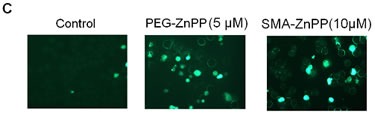

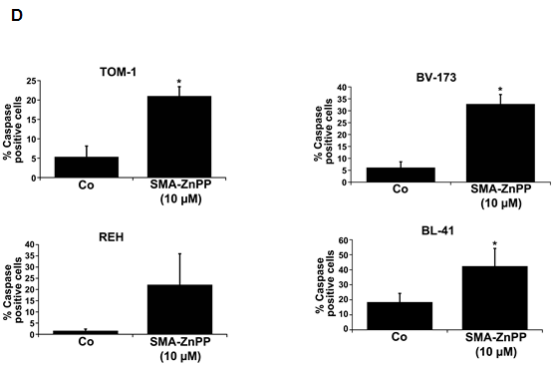
Hsp32-targeting drugs induce apoptosis in ALL cells A: Primary leukemic cells obtained from patient #17 with Ph^+^ ALL (imatinib-sensitive phase) and patient #18 with Ph^−^ ALL were incubated in control medium (Co) or medium containing increasing concentrations of either PEG-ZnPP or SMA-ZnPP at 37°C for 24 hours. Thereafter, the numbers of apoptotic cells were determined by light microscopy (upper panels) and AnnexinV staining and flow cytometry (lower panels). B: The lymphoid cells lines BV-173, TOM-1, REH and BL-41 were incubated in control medium (Co) or increasing concentrations of either SMA-ZnPP (left panel) or PEG-ZnPP (right panel) at 37°C for 48 hours. Then, the number (percentage) of apoptotic cells was determined by light microscopy. Asterisk (*): p<0.05. C: A Tunel assay was performed with Z-119 cells after incubation in control medium (Control), PEG-ZnPP (5 μM) or SMA-ZnPP (10 μM) at 37°C for 48 hours. After incubation, cells were analyzed under a fluorescence microscope and photographs taken as described in the text. Original magnification x 40. D: Flow cytometric evaluation of expression of active caspase 3 in two Ph^+^ALL cell lines (BV-173, TOM-1) and two Ph^−^ALL cell lines (REH, BL-41) after incubation in control medium or medium containing 10 μM of SMA-ZnPP. Results represent the mean±S.D. of three independent experiments.

Hsp32 has been described as a survival factor counteracting apoptosis in various neoplastic cells. We next investigated whether the growth-inhibitory effects of Hsp32 inhibitors (SMA-ZnPP and PEG-ZnPP) are associated with induction of apoptosis in ALL cells. We found that both drugs induce apoptosis in primary ALL cells and in the ALL cell lines tested (Figure 3 and Table 2). The apoptosis-producing effects of SMA-ZnPP and PEG-ZnPP on ALL cells were demonstrable by light microscopy (Figure 3A and 3B) as well as in a Tunel assay (Figure 3C). Furthermore, we were able to show by flow cytometry that SMA-ZnPP induces activation of caspase-3 in ALL cells (Figure 3D). In normal bone marrow cells, neither SMA-ZnPP nor PEG-ZnPP were found to induce apoptosis over the dose-range tested (1-40 μM) confirming previous data [29].

### Hsp32-targeting drugs synergize with BCR/ABL1-targeting drugs (imatinib, nilotinib) and with bendamustine in producing growth inhibition in ALL cells

**Figure4:**
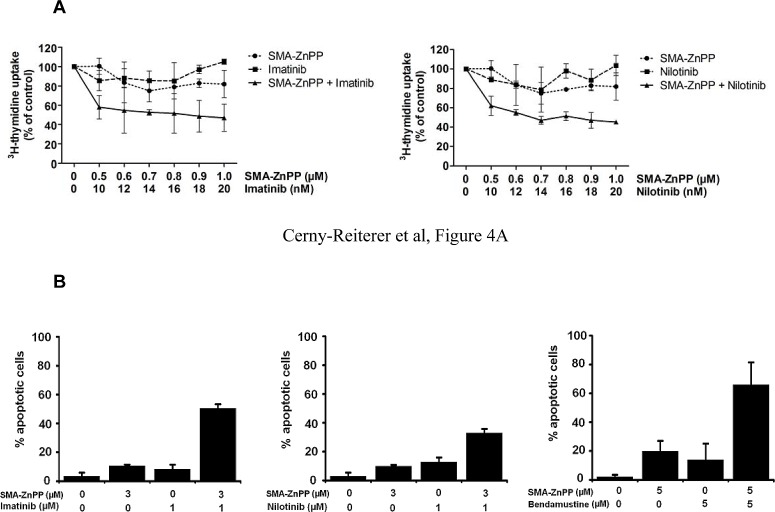

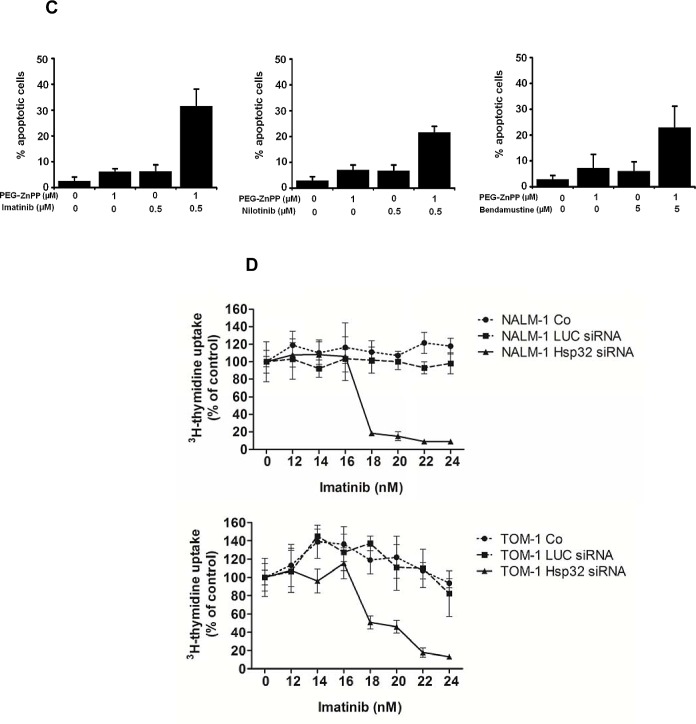
Hsp32-targeting drugs synergize with bendamustine and with BCR/ABL1-targeting drugs in producing growth inhibition and apoptosis in ALL cells A: The Ph^+^ ALL cell line TOM-1 was incubated in control medium or various concentrations of imatinib and SMA-ZnPP or a combination of both drugs for 48h. Thereafter, ^3^H-thymidine incorporation was measured. Results represent the mean±S.D. of triplicate experiments and are expressed in percent of control. B,C: The Ph^+^ ALL cell line Z-119 was incubated with control medium or various concentrations of imatinib, nilotinib, PEG-ZnPP, or SMA-ZnPP, alone or with a combination (with fixed drug ratio) of these drugs as indicated for 24 hours. Thereafter, the percentages of apoptotic cells were determined by light microscopy. Results represent the mean±S.D. of three independent experiments. D: The ALL cell lines NALM-1 and TOM-1 were left untransfected (Co) or were transfected with a control siRNA against luciferase (Luc siRNA) or siRNA against Hsp32 (200 nM each) as described in the text. 48 hours after transfection, cells were incubated in control medium or various concentrations of imatinib for 48h. Thereafter, ^3^H-thymidine incorporation was measured. Results represent the mean±S.D. of triplicate experiments and are expressed as percent of control.

Next, we examined cooperative drug effects on growth and survival (apoptosis) of ALL cells. We found that Hsp32-targeting drugs synergize with BCR/ABL1 TKI and with bendamustin in inducing growth inhibition and apoptosis in ALL cells (Figure 4A-C). To further validate Hsp32 as a potential drug-partner of TKI in ALL cells, we applied siRNA against Hsp32 and measured the proliferation of ALL cells as well as the response to imatinib. In these experiments, suboptimal concentrations of imatinib were applied. As shown in Figure 4D, addition of Hsp32-specific siRNA was found to potentiate the effects of imatinib on both ALL cell lines examined, TOM-1 and NALM-1.

## DISCUSSION

Recent data suggest that Hsp32 is an important survival factor and potential target in various malignant cells [22-27]. We have recently shown that CML cells constitutively express Hsp32 and that the disease-related oncoprotein BCR/ABL1 promotes expression of Hsp32 in Ba/F3 cells [28,29]. In the present study, we show that Hsp32 is also expressed and serves as an essential ´survival-molecule´ in Ph^+^ and Ph^−^ ALL cells. Our data also show that Hsp32-targeting drugs induce apoptosis and growth arrest in ALL cells and synergize with BCR/ABL1 TKI and with bendamustin in producing growth inhibition in imatinib-sensitive and imatinib-resistant ALL cells. Together, these data suggest that Hsp32 is a potential new target in ALL.

Expression of Hsp32 in ALL cells was demonstrable by qPCR and Western blotting as well as by immunocytochemistry. Interestingly, both the Ph^+^ ALL cells and Ph^−^ ALL cells were found to express Hsp32, suggesting that apart from BCR/ABL1, other mechanisms and molecules may also contribute to expression of this ´survival-molecule´ in leukemic cells. Baseline levels of Hsp32 were comparable in Ph^+^ and Ph^−^ ALL cells and were upregulated by hemin.

In Ph^+^ CML, BCR/ABL1 promotes the expression of Hsp32 in leukemic cells [28]. To explore the potential role of this pathway in expression of Hsp32 in Ph^+^ ALL cells, we treated these cells with BCR/ABL1-targeting drugs. We found that BCR/ABL1 TKI downregulate the expression of Hsp32 mRNA in Ph^+^ ALL cells. These data suggest that BCR/ABL1 may contribute to expression of Hsp32 in Ph^+^ ALL cells. However, as mentioned, Hsp32 was also detectable in Ph^−^ ALL cells. From these data, we hypothesize that expression of Hsp32 can also be triggered by other pathways in ALL cells. Indeed it has been described that several different oncoproteins, including JAK2 V617F, KIT D816V or RAS G12V induce expression of Hsp32/HO-1 in neoplastic cells [30]. The exact nature of additional HO-1-promoting oncogenic lesions in ALL cells remains at present unknown. Alternatively, Hsp32 expression in ALL cells may also be regulated by external factors. In this regard it is noteworthy that Hsp32 is an established “stress-induced” survival factor in various physiologic cells and that several different stimuli, including chemotherapy agents, can induce expression of Hsp32/HO-1 in malignant cells [30].

To demonstrate that Hsp32 serves as a survival factor in ALL cells, we performed experiments using ALL cell lines and siRNA against Hsp32. The observation that the siRNA-induced knock-down is associated with apoptosis and growth arrest in these cells suggests that Hsp32 serves as an important survival factor in ALL cells and thus may represent a potential therapeutic target.

In the past few years, two water-soluble pharmacologic inhibitors of Hsp32, SMA-ZnPP and PEG-ZnPP, have been developed and tested in experimental solid tumors [22-27]. In the current study these two inhibitors were applied to target Hsp32 in ALL cells. Both inhibitors were found to downregulate growth and survival in primary (Ph^+^ and Ph^−^) ALL cells as well as in all ALL cell lines tested. By contrast, the Hsp32 inhibitors showed no major effects on viability of normal cells [28]. All in all, these data suggest that pharmacologic targeting of Hsp32 in ALL cells may result in their selective apoptosis and growth arrest.

A major clinical challenge in the treatment of ALL is resistance to imatinib and other BCR/ABL1 TKI [10-13]. Therefore, a number of novel agents and pharmacologic approaches are currently under investigation, with the aim to overcome drug-resistance. In the present study, we found that PEG-ZnPP and SMA-ZnPP induce growth arrest and apoptosis not only in imatinib-sensitive ALL cells but also in imatinib-resistant cells, which may be of clinical interest. These data are also in line with our previous observations that Hsp32 inhibitors block the growth of imatinib-resistant CML cells as well as Ba/F3 cells expressing various imatinib-resistant mutants of BCR/ABL1, including the T315I mutant that renders BCR/ABL1 resistant against all currently available BCR/ABL1 TKI [29]. In the present study, we examined one CML patient in lymphoid blast phase exhibiting the T315I mutant. As expected, both PEG-ZnPP and SMA-ZnPP were found to suppress the growth of leukemic cells in this patient. Together, our data suggest that targeting of Hsp32 may be an interesting approach to treat patients with drug-resistant Ph^+^ ALL or lymphoid blast phase of CML.

An attractive strategy to overcome drug resistance in leukemias is to combine various targeted drugs with each other or with conventional drugs. The data of our study show that Hsp32-targeting drugs synergize with imatinib and with nilotinib as well as with bendamustine in producing growth inhibition in Ph^+^ and Ph^−^ ALL cells. These synergistic drug effects were seen in imatinib-sensitive as well as in imatinib-resistant ALL cells, supporting the notion that Hsp32 may be an attractive new therapeutic target in this disease. This hypothesis was further supported by the observation that siRNA against HO-1 substantially augments the growth-inhibitory effects of imatinib on ALL cells.

In summary, our data show that Hsp32 is an important survival factor and potential new target in leukemic cells in Ph^+^ and Ph^−^ ALL, including patients with TKI-resistant disease. Clinical studies are now warranted to show whether targeting of Hsp32 alone or in combination with BCR/ABL1 TKI or other inhibitors, can induce clinically relevant responses in patients.

## MATERIALS AND METHODS

### Reagents

Pegylated zinc protoporphyrin (PEG-ZnPP) and ZnPP encapsulated in the micelle of styrene maleic acid (SMA-ZnPP), were produced as described previously [31-33]. Imatinib and nilotinib (AMN107) were kindly provided by Dr.E.Buchdunger and Dr.P.W.Manley (Novartis Pharma AG, Basel, Switzerland). Bendamustine was kindly provided by Dr.D.Guggi (Mundipharma, Vienna, Austria). RPMI 1640 medium and fetal calf serum (FCS) were purchased from PAA laboratories (Pasching, Austria), hemin and bovine serum albumin (BSA) from Sigma-Aldrich (St. Louis, Mo), and lipofectin from Invitrogen (Carlsbad, CA).

### Primary ALL cells and cell lines

For *in vitro* culture experiments, primary leukemic cells were obtained from 11 patients with Ph^+^ ALL, 15 with Ph^−^ ALL, 4 with T-ALL, 2 with biphenotypic acute leukemia, and one with a lymphoid blast phase of CML with *BCR/ABL1* T315I. For polymerase chain reaction (PCR) analysis, frozen samples from 10 patients with Ph^+^ ALL and 10 with Ph^−^ ALL were used. In 8 patients (5 with Ph+ ALL and 3 with Ph- ALL), CD34^+^/CD38^−^ cells and CD34^+^/CD38^+^ cells were purified by cell sorting (purity >98%) as described [34]. The patients´ characteristics are shown in Table 1. Written informed consent was obtained in each case. The study was approved by the Ethics Committee of the Medical University of Vienna, Austria. The following Ph^+^ ALL cell lines were used: Z-119, BV-173, TOM-1, and NALM-1. In addition, a number of Ph^−^ lymphatic cell lines were used: Raji, Ramos, REH, and BL-41. Z-119 cells were kindly provided to J.V.M. by Dr. Zeev Estrov (MD Anderson Cancer Centre, Houston, Texas, USA). All other cell lines were purchased from the Leibnitz Institute DSMZ-German Collection of Microorganisms and Cell Cultures (Braunschweig, Germany). The identity of the cell lines was reconfirmed by DNA sequencing and DNA profiling (by nonaplex PCR) and by studying the presence or absence of *BCR/ABL1*. Cell lines were cultured in RPMI 1640 medium and 20% heat-inactivated FCS at 37°C and 5% CO_2_. Table 2 shows a summary of characteristics of cell lines tested in this study.

### Real time PCR

RNA was isolated from primary ALL cells and cell lines using the RNeasy MinEluteCleanupKit (Qiagen, Hiden, Germany). cDNA was synthesized using Moloney murine leukemia virus reverse transcriptase (Invitrogen), random primers, First Strand buffer, dNTPs (100 mM), and RNasin (all from Invitrogen) according to the manufacturer`s instructions. PCR was performed using primers specific for Hsp32 and ABL: Hsp32: 5´-CAGGATTTGTCAGAGGCC CTGAAGG-3´ (forward), 5´-TGTGGTACAGGGAGGCCATCACC-3´ (reverse); ABL: 5´-TGTATGATTTTGTGGCCAGTGGAG-3´ (forward), and 5´-GCCTA AGACCCGGAGCTTTTCA-3´ (reverse). mRNA levels were quantified on a 7900HT Fast Real-Time PCR System (Applied Biosystem, Foster City, CA) using iTAq SYBR Green Supermix with ROX (Bio-Rad, Hercules, CA). Hsp32 mRNA expression levels were normalized to ABL mRNA levels. Calculations were based on standard curves established for Hsp32 and ABL mRNA expression. Hsp32 mRNA levels were expressed as percentage of ABL mRNA.

### Immunocytochemistry

Immunocytochemistry was performed on cytospin-slides prepared with primary neoplastic cells, sorted ALL stem cells, and cell lines. A polyclonal rabbit anti- HO-1 (anti-Hsp32) antibody (Stressgen, Ann Arbor, MI; dilution 1:100) and a biotinylated goat-anti-rabbit IgG (Biocare, San Diego, CA) were applied essentially as described [28,35]. Slides were incubated with the primary antibody overnight, washed, and were then incubated with second-step antibody for 30 minutes. Streptavidin-alkaline-phosphatase complex (Biocare) was used as chromogen. Antibody reactivity was made visible using Neofuchsin (Nichirei, Tokyo, Japan). Slides were counterstained in Mayer`s hemalaun. In control experiments, the primary antibody was preincubated with control buffer or a Hsp32-specific blocking peptide (Stressgen) before being applied.

### Western blotting

The Ph^+^ cell line Z-119 and the Ph^−^ cell line BL-41 were incubated with hemin (10 μM, 37°C, 8 hours) before being analyzed. Western blotting was performed using a polyclonal rabbit anti-Hsp32 antibody (Stressgen) and an anti--actin antibody (Santa Cruz), as described [28,35]. Antibody reactivity was made visible by donkey anti-rabbit IgG antibody and Lumigen PS-3 detection reagent (both from GE Healthcare, Buckinghamshire, UK).

### Design and application of siRNA against Hsp32

siRNA against Hsp32 (5'-AAGCUUUCUGGUGGCGACAGUdTdT-3') as well as a control siRNA against luciferase (5'-CUUACGCUGAGUACUUCG AdTdT-3') were synthesized by Dharmacon Research (Lafayette, CO). The siRNA (200 nM) was transfected into NALM-1, Raji and TOM-1, using lipofectin (Invitrogen) as reported [35]. Proliferation of transfected and control cells was analyzed by determining ^3^H-thyimidine uptake. In addition, the percentage of apoptotic cells was determined by Wright-Giemsa staining after 48 hours. In a separate set of experiments, siRNA-transfected NALM-1 and TOM-1 cells were incubated in various concentrations of imatinib (10-24 nM) at 37°C for 48 hours. Then, cells were examined for proliferation by measuring ^3^H-thyimidine uptake.

### Proliferation assay

To examine anti-proliferative effects of PEG-ZnPP and SMA-ZnPP, Ph^+^ and Ph^−^ ALL cell lines and primary ALL cells were cultured in 96-well microtiter plates (5 x 10^4^ cells per well) in the absence or presence of various concentrations of PEG-ZnPP or SMA-ZnPP for 48 hours, followed by addition of ^3^H-thymidine (0.5 μCi per well) for 16 hours. Cells were harvested on filter membranes (Packard Bioscience, Meriden, CT) in a Filtermate 96 harvester (Packard Bioscience). Filters were air-dried, and the bound radioactivity was measured in a -counter (Top-Count NXT, Packard Bioscience). All experiments were performed in triplicate. In a separate set of experiments, cell lines were cultured in the presence of various drug combinations at a fixed concentration-ratio for each combination: PEG-ZnPP+imatinib, PEG-ZnPP+nilotinib, PEG-ZnPP+bendamustine, SMA-ZnPP+imatinib, SMA-ZnPP+nilotinib, and SMA-ZnPP+bendamustine.

### Apoptosis assays

The effects of SMA-ZnPP and PEG-ZnPP on cell viability (apoptosis) were analyzed by morphologic examination. Cells were incubated with various concentrations of SMA-ZnPP or PEG-ZnPP (1-20 μM) at 37°C for 48 hours. The percentage of apoptotic cells was quantified on Wright-Giemsa-stained cytospin slides [36]. In select experiments, Hsp32-targeting drugs were applied in combination with TKI (imatinib or nilotinib) or bendamustine. To confirm apoptosis in drug-exposed cells, combined AnnexinV/propidium-iodide staining was performed using the apoptosis detection kit from Alexis Biochemicals (Lausen, Switzerland) as described [29]. Cells were analyzed by flow cytometry on a FACScan (Becton Dickinson, San Jose, CA). A Tunel assay was performed using the ´In situ cell death detection kit´ (Roche, Mannheim, Germany) as reported [35]. In brief, cells were incubated with 10 μM SMA-ZnPP, 5 μM PEG-ZnPP, or control medium for 48 hours and then spun on cytospin slides, fixed in 4% paraformaldehyde, washed, and permeabilized in 0.1% Triton X-100 and 0.1 % sodium citrate. Then, cells were washed and incubated in terminal-transferase reaction-solution for 60 minutes at 37°C. Cells were analyzed under a Carl Zeiss Imager. A1 microscope (Carl Zeiss, Jena, Germany). For caspase 3 detection, cell lines were incubated in control medium or in various concentrations of SMA-ZnPP (5-20 μM) at 37°C for 48 hours. Then, cells were fixed in 2% formaldehyde (room temperature, 10 minutes), permeabilized in 100% methanol at -20°C (15 minutes), washed in PBS plus BSA (0.1%), and then stained with the FITC-conjugated mAb C92-605 (Becton Dickinson Biosciences) directed against active caspase 3 for 1 hour. Thereafter, cells were analyzed by flow cytometry on a FACSCalibur (Becton Dickinson Biosciences).

### Statistical analysis

The paired Student´s t test was applied in growth inhibition-experiments. Results were considered to be significantly different, when the p-value was <0.05. Drug combination effects (additive versus synergistic) were determined by calculating the combination index (CI) values using Calcusyn software (Calcusyn; Biosoft, Ferguson, MO) [37]. A CI value of 1 indicates additive effects and a CI below 1 indicates synergistic drug interactions.
